# Local delivery of novel MRTF/SRF inhibitors prevents scar tissue formation in a preclinical model of fibrosis

**DOI:** 10.1038/s41598-017-00212-w

**Published:** 2017-03-31

**Authors:** Cynthia Yu-Wai-Man, Bradley Spencer-Dene, Richard M. H. Lee, Kim Hutchings, Erika M. Lisabeth, Richard Treisman, Maryse Bailly, Scott D. Larsen, Richard R. Neubig, Peng T. Khaw

**Affiliations:** 1 UCL Institute of Ophthalmology, London, UK; 2 National Institute for Health Research (NIHR) Biomedical Research Centre at Moorfields Eye Hospital NHS Foundation Trust and UCL Institute of Ophthalmology, London, UK; 3 Signalling and Transcription Group, Francis Crick Institute, London, UK; 4 Experimental Histopathology STP, Francis Crick Institute, London, UK; 5 Vahlteich Medicinal Chemistry Core, College of Pharmacy, University of Michigan, Ann Arbor, MI USA; 6 Department of Pharmacology and Toxicology, Michigan State University, East Lansing, MI USA

## Abstract

The myocardin-related transcription factor/serum response factor (MRTF/SRF) pathway represents a promising therapeutic target to prevent fibrosis. We have tested the effects of new pharmacological inhibitors of MRTF/SRF signalling in a preclinical model of fibrosis. CCG-222740, a novel MRTF/SRF inhibitor, markedly decreased SRF reporter gene activity and showed a greater inhibitory effect on MRTF/SRF target genes than the previously described MRTF-A inhibitor CCG-203971. CCG-222740 was also five times more potent, with an IC_50_ of 5 μM, in a fibroblast-mediated collagen contraction assay, was less cytotoxic, and a more potent inhibitor of alpha-smooth muscle actin protein expression than CCG-203971. Local delivery of CCG-222740 and CCG-203971 in a validated and clinically relevant rabbit model of scar tissue formation after glaucoma filtration surgery increased the long-term success of the surgery by 67% (*P* < 0.0005) and 33% (*P* < 0.01), respectively, and significantly decreased fibrosis and scarring histologically. Unlike mitomycin-C, neither CCG-222740 nor CCG-203971 caused any detectable epithelial toxicity or systemic side effects with very low drug levels measured in the aqueous, vitreous, and serum. We conclude that inhibitors of MRTF/SRF-regulated gene transcription such as CCG-222740, potentially represent a new therapeutic strategy to prevent scar tissue formation in the eye and other tissues.

## Introduction

Fibrosis represents one of the largest unmet needs in clinical medicine and accounts for over 40% of all deaths. In the eye, fibrosis is also linked to the pathogenesis or failure of treatment of most blinding diseases worldwide. Glaucoma is the leading cause of irreversible blindness and its prevalence is estimated to reach 79.6 million people by 2020, with more than 11 million individuals suffering from bilateral blindness^1^. Wound contraction and scarring are the principal causes of blockage of aqueous flow at the drainage site in glaucoma filtration surgery^2^. Current clinical practice to counteract these phenomena uses the cytotoxic antimetabolites mitomycin-C (MMC) and 5-fluorouracil (5-FU). These non-specific agents cause widespread cell death and apoptosis^3^ and have long-standing effects on cell proliferation^4^. Their use is also associated with potentially blinding complications, such as tissue breakdown and infection^5, 6^. Alternative agents with more targeted physiological effects and less cytotoxicity are thus needed.

SRF is a ubiquitous transcription factor that was first identified as a regulator of growth-factor regulated immediate-early genes. Working in partnership with its coactivators MRTF-A and MRTF-B, SRF regulates the majority of cytoskeletal genes^7, 8^, including many that are involved in fibrosis^9–11^. The activity of MRTF-A and MRTF-B, which bind G-actin through N-terminal RPEL motifs, responds to variations in the cellular concentration of G-actin induced by Rho GTPase signalling^12, 13^. Given the potential connection between MRTF/SRF signalling and fibrosis, there has been considerable interest in exploiting it as a route to clinical intervention and studies have previously been undertaken to evaluate this in mouse models of vascular^9^, skin^10^, and lung fibrosis^11^.

We previously sought to identify inhibitors of MRTF-SRF signalling by high-throughput screening using an MRTF-SRF reporter gene that selectively responds to RhoA-induced gene transcription^14^. Using this approach, we identified CCG-1423 as a potent inhibitor of RhoA-stimulated MRTF/SRF gene transcription^15^. More recently, we have also optimised a second generation inhibitor, CCG-203971, which is less cytotoxic than CCG-1423^16, 17^. The mechanism of action of these compounds remains unclear: it has been proposed that CCG-1423 is an inhibitor of MICAL-2, a member of the MICAL family of actin-binding mono-oxygenases^18^, or that it acts directly on MRTF-A and other RPEL-family of proteins to prevent the nuclear import of MRTF-A^19^.

Here we investigate the potential local application of MRTF/SRF inhibitors to counter fibrosis in the eye and other tissues^20, 21^. We show that a novel second-generation MRTF/SRF inhibitor, CCG-222740, exerts a greater inhibitory effect on MRTF/SRF target genes than CCG-203971. CCG-222740 is also more potent at preventing alpha-smooth muscle actin protein expression, is less cytotoxic, and effectively prevents scar tissue formation in a preclinical model of fibrosis.

## Results

### CCG-222740 is a novel and effective MRTF/SRF pathway inhibitor

**Figure 1:**
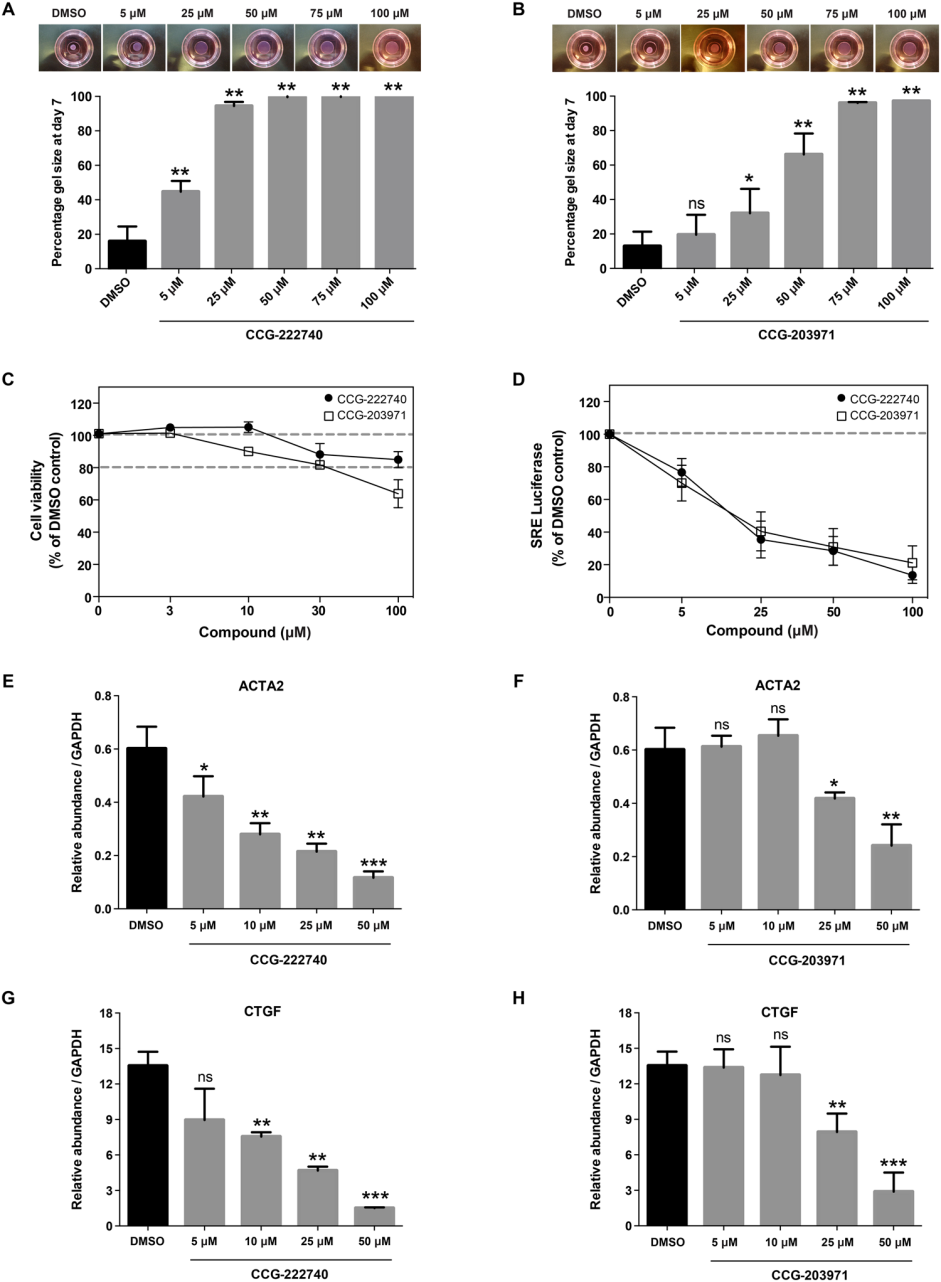
CCG-222740 is an effective MRTF/SRF pathway inhibitor. (**A**,**B**) Human conjunctival fibroblasts were seeded in collagen type I matrix and cultured for 7 days with or without CCG-222740 and CCG-203971 at the indicated concentrations. Each experiment used triplicate gels per condition, and the experiment was repeated three times; (**C**) Effect of CCG-222740 and CCG-203971 in cell proliferation, assessed using CellTiter 96 Aqueous one solution assay. Results are mean ± SEM for triplicate experiments; (**D**) Human conjunctival fibroblasts were transfected with the SRF reporter gene 3DA.Luc, serum-starved, and treated with different concentrations of CCG-222740 or CCG-203971 or 0.1% DMSO control for 24 hours. Results represent mean ± SEM for triplicate experiments; (**E**,**F**,**G**,**H**) Human conjunctival fibroblasts were treated with different concentrations of CCG-222740 or CCG-203971 or 0.1% DMSO control in 10% FCS + DMEM for 24 hours, and MRTF/SRF target gene expression assessed by qRT-PCR. Results represent mean ± SEM for triplicate experiments. *P < 0.05; **P < 0.01; ***P < 0.001.

**Figure 2:**
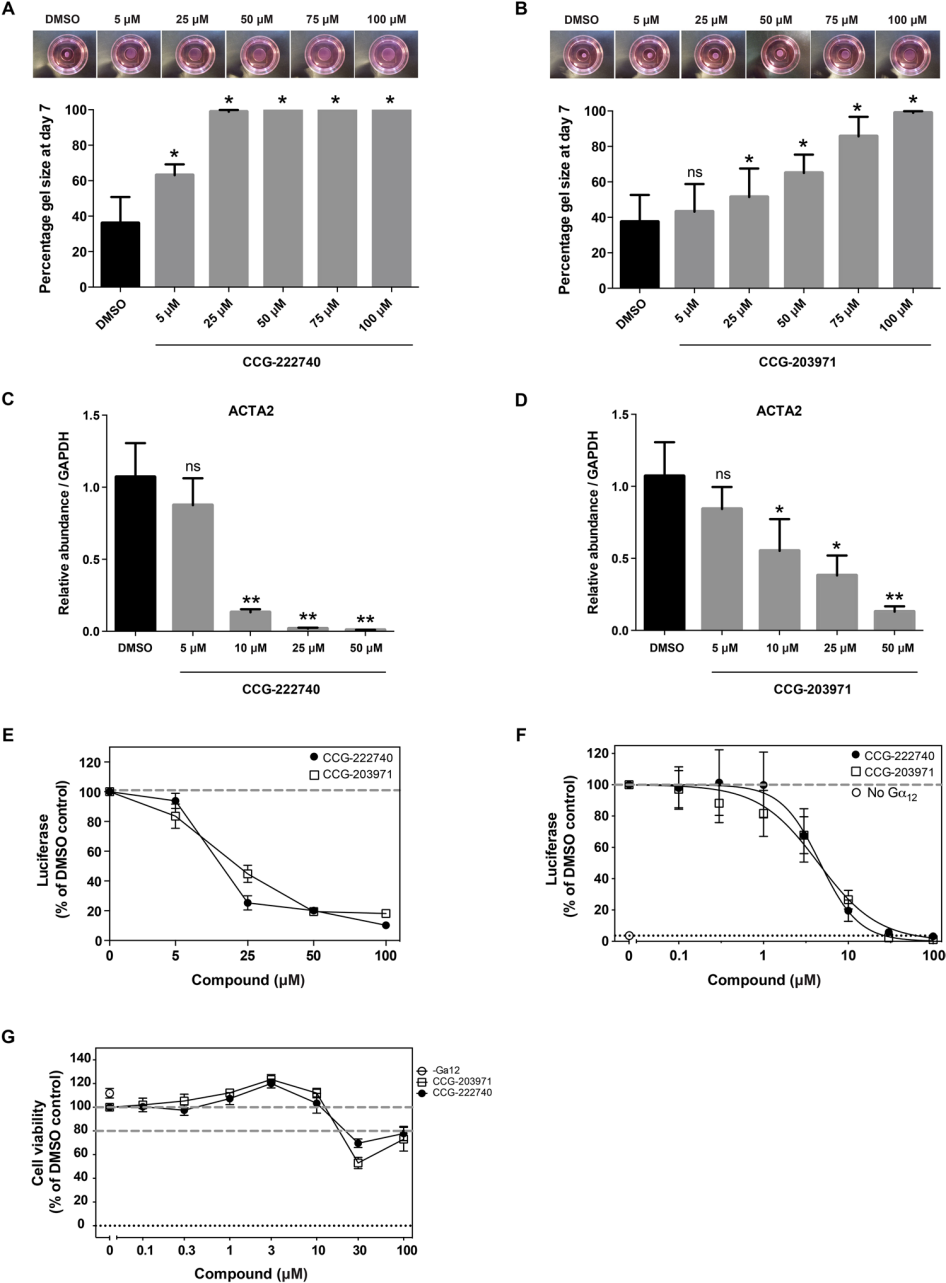
CCG-222740 is more effective than CCG-203971 in rabbit conjunctival fibroblasts. (**A**,**B**) Rabbit conjunctival fibroblasts were seeded in collagen type I matrix and cultured for 7 days as in Fig. 1A and B. (**C**,**D**) Analysis of *ACTA2* gene expression in rabbit conjunctival fibroblasts was analysed by qRT-PCR as in Fig. 1E and F. Results represent mean ± SEM for triplicate experiments. *P < 0.05; **P < 0.01. (**E**) Rabbit conjunctival fibroblasts were transfected with the SRF reporter 3DA.luc, serum-starved, and treated with different concentrations of CCG-222740 or CCG-203971 or 0.1% DMSO control for 24 hours. Results represent mean ± SEM for triplicate experiments; (**F**) HEK293 cells were transfected with the SRF reporter SRE.L luc and treated as in (**E**). Results represent mean ± SEM for triplicate experiments; (**G**) HEK293 cell viability in the presence of CCG-222740 or CCG-203971 was measured using the WST-1 assay. Results represent mean ± SEM for triplicate experiments.

We tested two candidate MRTF/SRF pathway inhibitors, CCG-222740 and CCG-203971, using a functional three-dimensional fibroblast-populated collagen contraction assay. We chose this assay as it has been shown to be a good *in vitro* model to study tissue contraction^22–24^. CCG-222740 and CCG-203971 both decreased collagen matrix contraction in a concentration-dependent manner but CCG-222740 was five times more potent than CCG-203971 in human conjunctival fibroblasts [IC_50_ = 5 μM compared to 25 μM] (Fig. 1A and B). We also confirmed our results in rabbit conjunctival fibroblasts, where CCG-222740 was similarly more effective at decreasing collagen matrix contraction than CCG-203971 (Fig. 2A and B). In addition, CCG-222740 was less cytotoxic than CCG-203971 with a cell viability of 100% at 10 μM, 88% at 30 μM, and 85% at 100 μM (Fig. 1C).

We next compared how CCG-222740 and CCG-203971 affect the activity of an SRF reporter gene in transfected cells. Both compounds showed similar activity, with almost 100% inhibition of baseline reporter activity (Figs 1D and 2E,F). However, examination of endogenous gene expression revealed that CCG-222740 was a more potent inhibitor of expression for two MRTF/SRF target genes classically linked to fibrosis (*ACTA2*, coding for alpha-smooth muscle actin – αSMA; and *CTGF*, coding for Connective Tissue Growth Factor)^25, 26^ (Fig. 1E–H). CCG-222740 also decreased *ACTA2* gene expression to a greater extent than CCG-203971 in rabbit conjunctival fibroblasts (Fig. 2C and D).

**Figure 3:**
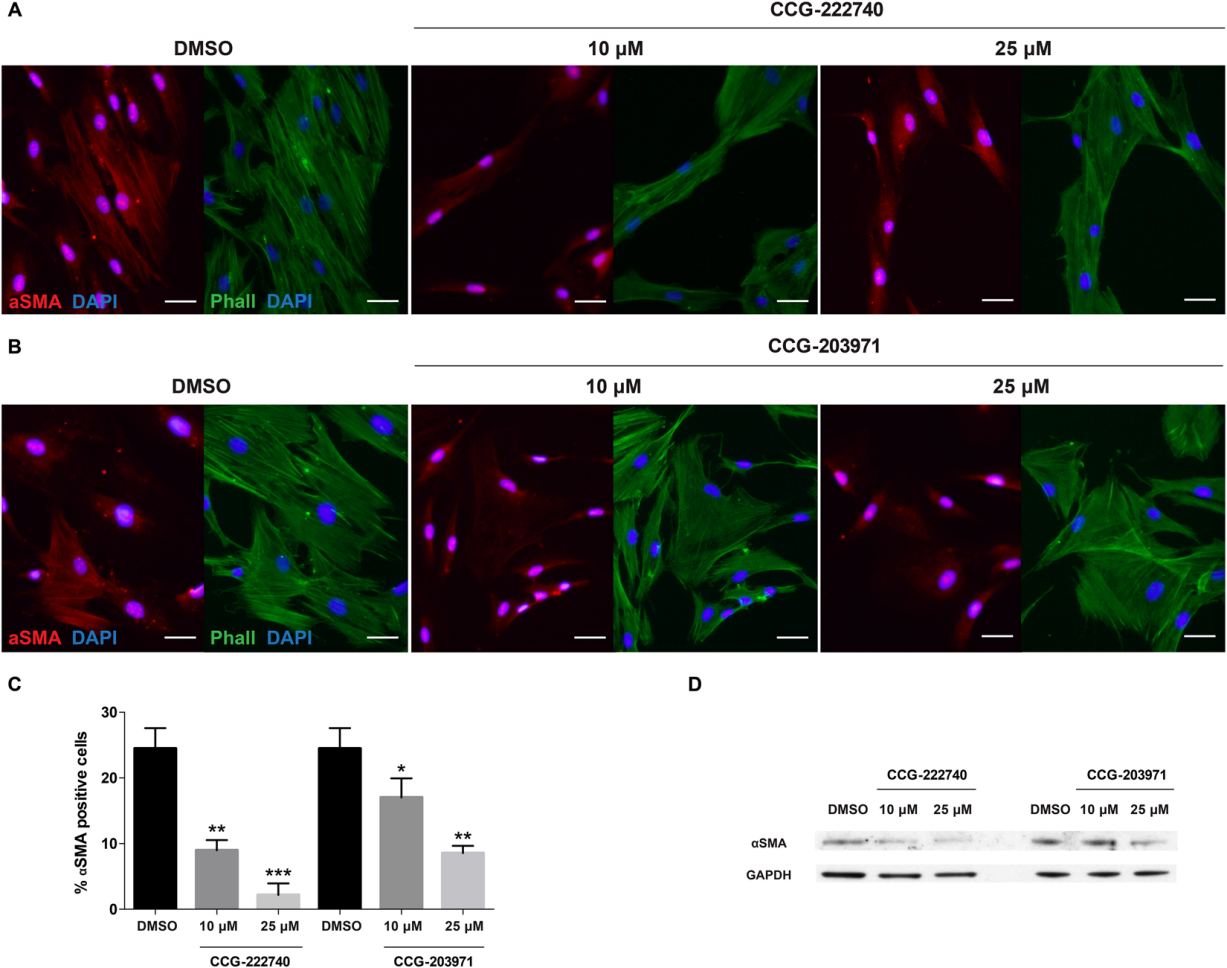
CCG-222740 is more potent than CCG-203971 at decreasing αSMA protein expression. (**A**,**B**) Human conjunctival fibroblasts were treated for 48 hours with 10 or 25 μM of CCG-222740 or CCG-203971 or 0.1% DMSO control in DMEM +10% FCS. The fibroblasts were stained with Cy3-labelled alpha smooth muscle actin antibody, phalloidin and DAPI. Scale bar = 30 μm; (**C**) Fibroblasts that are positive for αSMA clearly display αSMA-positive stress fibres, which were quantified visually from three random fields of view per treatment group. Results represent mean ± SEM for triplicate experiments. *P < 0.05; **P < 0.01; ***P < 0.001. (**D**) Western blot analysis of αSMA protein expression in human conjunctival fibroblasts treated with CCG-222740 or CCG-203971 as in (**A**,**B**). Cropped blots are displayed and the full-length blots are shown in Supplementary Figure S1.

We further tested the effects of the inhibitors on αSMA protein expression and found that CCG-222740 was more potent than CCG-203971 at decreasing αSMA protein expression in human conjunctival fibroblasts (Fig. 3D). Human conjunctival fibroblasts grown in 10% serum contain a small subpopulation of fibroblasts with prominent αSMA-positive stress fibres (about 20%). CCG-222740 treatment decreased the percentage of αSMA-positive fibroblasts from 24.5% to 9.0% (*P* < 0.01) and 2.2% (*P* < 0.001) at 10 μM and 25 μM, respectively (Fig. 3A and C). CCG-203971 treatment only decreased the percentage of αSMA-positive fibroblasts from 24.5% to 17.0% (*P* < 0.05) and 8.6% (*P* < 0.01) at 10 μM and 25 μM, respectively (Fig. 3B and C).

### CCG-222740 and CCG-203971 increase the long-term success of surgery in a preclinical model of fibrosis

We next studied the effects of CCG-222740 and CCG-203971 using a validated and clinically relevant animal model of wound healing after glaucoma filtration surgery^27, 28^. Subconjunctival scarring after glaucoma filtration surgery was chosen as it is one of the most aggressive models of scar tissue formation, and failure of surgery is due to excessive scarring. This animal model is also validated for scar tissue formation in humans and agents that have reduced scarring in the rabbit have been shown to be effective in humans in clinical trials^29–31^.

Drugs are rapidly cleared from the subconjunctival space after glaucoma filtration surgery^32^, and we therefore adopted a dosing strategy in which subconjunctival injections into the bleb were administered daily for the first nine days followed by twice weekly injections for three weeks. This protocol, previously used in the rabbit model for anti-scarring inhibitors requiring continuous exposure^4^, maximises local availability of the drug at the critical time of postoperative wound repair, and maintains availability during the later stages of wound remodelling^33^ (see Materials and Methods). CCG-222740 and CCG-203971 were used at concentrations similar to the serum concentrations resulting from systemic administration (100 mg/kg) in mouse models of dermal and lung fibrosis^10, 11^.

**Figure 4:**
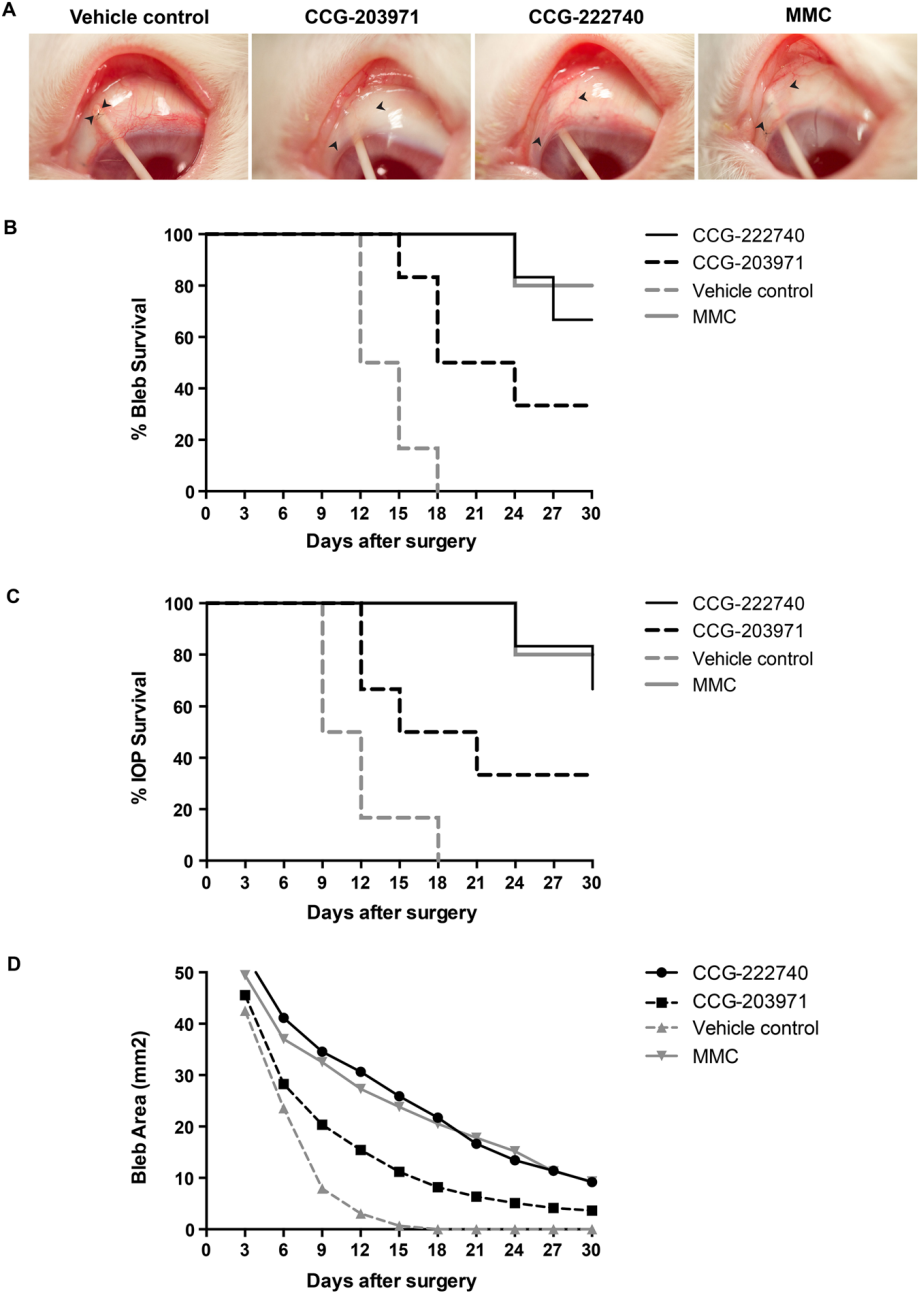
CCG-222740 and CCG-203971 increase the long-term success of surgery in a preclinical model of fibrosis. (**A**) Morphology of blebs after surgery and treatment with CCG-222740, CCG-203071, MMC or vehicle. Arrows indicate bleb edges. (**B**) Kaplan-Meier graph comparing the bleb survival between CCG-222740 [N = 6], CCG-203971 [N = 6], MMC [N = 6], and vehicle control [N = 6]. CCG-222740 and CCG-203971 treatments significantly increased bleb survival by 67% (*P* < 0.0005) and 33% (*P* < 0.01), respectively, compared to vehicle control. (**C**) Intraocular pressure (IOP) survival, i.e. when the intraocular pressure remains below the baseline IOP measurement. CCG-222740 and CCG-203971 treatments increased IOP survival by 67% (*P* < 0.0005) and 33% (*P* < 0.05), respectively. (**D**) Comparison of bleb areas.

A bleb arises when a filtration cannula is inserted into the eye during the surgery and drains aqueous fluid from the anterior chamber of the eye to under the conjunctival tissue, forming a visible raised collection of fluid. We used bleb survival (fluid present under the conjunctival tissue) as our primary end-point as this is indicative of a significant reduction in scar tissue formation and the long-term opening of the pathway created during the surgery. A bleb is considered to have failed when it becomes flat, scarred, vascularised and is associated with a deep anterior chamber. At day 30, the blebs that survived after treatment with CCG-222740 or CCG-203971 remained diffusely elevated whereas all the blebs in animals treated with vehicle control became flat, scarred and vascularised (Fig. 4A). CCG-222740 and CCG-203971 treatments increased the long-term success of the surgery by 67% (*P* < 0.0005) and 33% (*P* < 0.01), respectively, compared to vehicle control (Fig. 4B). When compared to the cytotoxic antimetabolite, mitomycin C (MMC), CCG-222740 had equally good surgical outcomes with the blebs failing at a mean of 28.5 days (SEM 1.0) with CCG-222740 and 28.8 days (SEM 1.2) with MMC. CCG-203971 had a worse surgical outcome compared to CCG-222740 with the blebs failing earlier at 22.5 days (SEM 2.7), but it was better than the vehicle control-treated rabbits where all the blebs failed before day 30 and the mean failure day was 14 days (SEM 1.0).

We used intraocular pressure (IOP) survival and bleb area as secondary end-points in the study. IOP is considered to have failed when there is an indefinite return to baseline level or greater. IOP was significantly lower in the MMC-treated group (*P* < 0.002) compared to vehicle control. IOP also remained consistently lower than baseline measurements in the groups treated with CCG-222740 (*P* < 0.0005) and CCG-203971 (*P* < 0.05) (Fig. 4C). In addition, the CCG-222740-treated group had significantly larger bleb area (*P* < 0.02) compared to vehicle control (Fig. 4D). The bleb area was also significantly larger in the group treated with MMC (*P* < 0.02) compared to vehicle control. There were, however, no statistically significant differences in the bleb area between the CCG-203971-treated group and vehicle control. Taken together, these results show that inhibition of MRTF/SRF signalling is effective at increasing the long-term success of surgery in this preclinical model of fibrosis, and that CCG-222740 is more effective than CCG-203971.

### Local delivery of CCG-222740 or CCG-203971 prevents scar tissue formation

**Figure 5:**
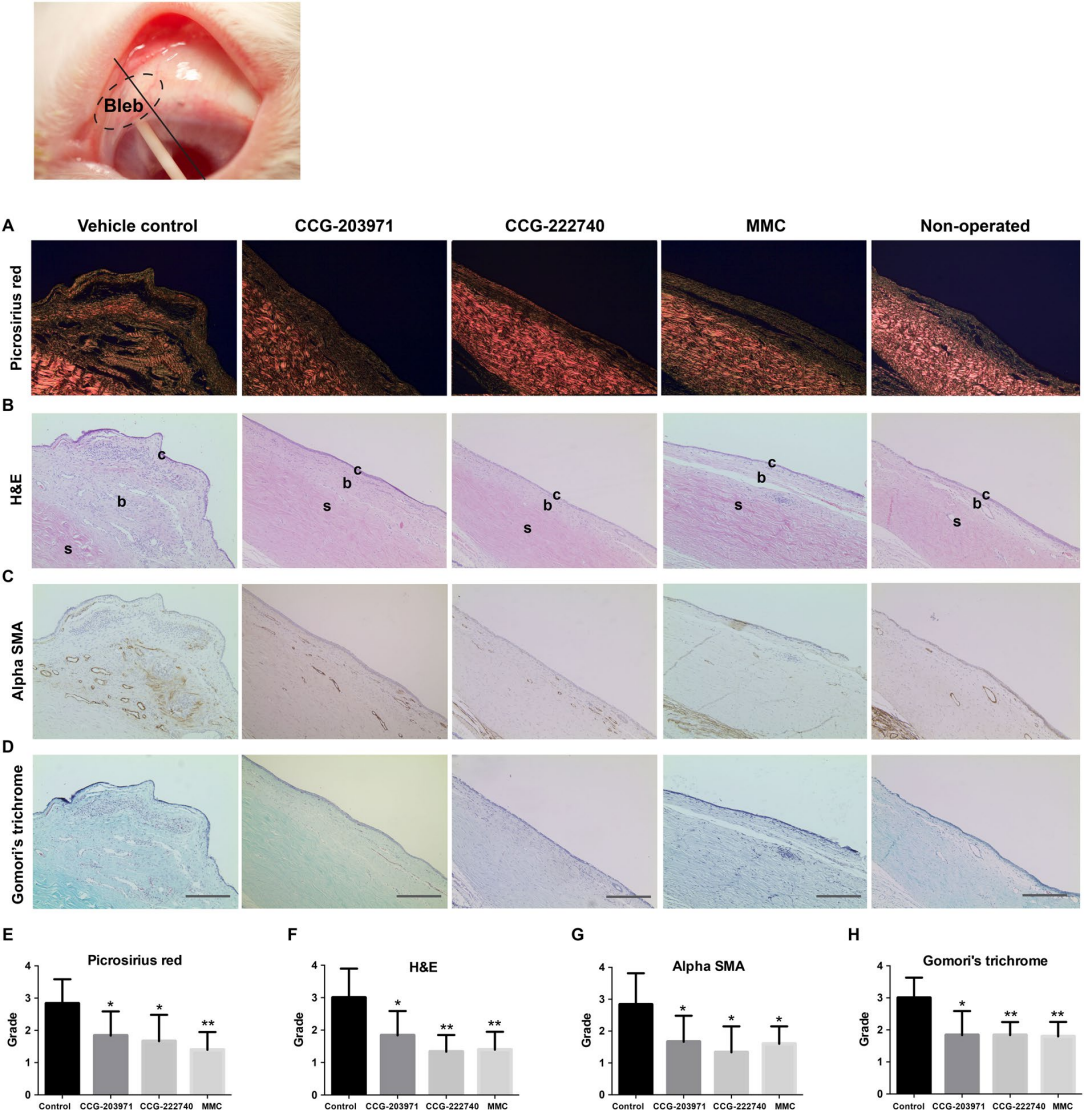
Local delivery of CCG-222740 or CCG-203971 prevents scar tissue formation. The white filtration tube was used as landmark, and tissue was sectioned through the bleb area on a plane 2 mm from the tube and stained (black line). The left operated eyes were compared to the right non-operated eyes, which were used as controls for the appearance of normal conjunctival tissue. Scale bar = 100 μm; c, conjunctiva, b, subconjunctival space, s, sclera. Analysis was performed using the mean results in each treatment group. Statistically significant differences are expressed as *P < 0.05; **P < 0.01. (**A**,**E**) Picrosirius red; (**B**,**F**) H&E; (**C**,**G**) αSMA; (**D**,**H**) Gomori’s trichrome stain for extracellular matrix and collagen.

We further evaluated the histologic differences after local delivery of CCG-222740 and CCG-203971. The white filtration tube was used as landmark and the plane of sectioning of the bleb area was performed 2 mm from the tube (black line in Fig. 5). Grading was performed using a modified system originally described by Shah *et al*.^34^. The left operated eyes were compared to the right non-operated eyes that were used as controls for the appearance of normal conjunctival tissue. The sections were graded using a scale of −4 to +4 (0 = same as control eye; 1 = 1–25% difference from control eye; 2 = 26–50% difference from control eye; 3 = 51–75% difference from control eye; 4 = >75% difference from control eye; prefix+, more than; prefix−, less than). Analysis was performed using the mean results in each treatment group.

There were some variations between conjunctival sections from different rabbits but a significant decrease in scar tissue formation was noted after treatment with CCG-222740 (*P* < 0.03) or CCG-203971 (*P* < 0.05), compared to vehicle control (Fig. 5A and E). Total cellularity also remained significantly increased in the vehicle-treated group compared to the CCG-222740-treated (*P* < 0.005) or CCG-203971-treated groups (*P* < 0.05) (Fig. 5B and F). In addition, CCG-222740 (*P* < 0.02) or CCG-203971 treatments (*P* < 0.05) significantly decreased the expression of αSMA by cells, suggesting the presence of fewer myofibroblasts (Fig. 5C and G). The presence of newly laid extracellular matrix and collagen was also present to a greater degree in the vehicle-treated group compared to the CCG-222740-treated (*P* < 0.005) or CCG-203971-treated groups (*P* < 0.02) (Fig. 5D and H).

### CCG-222740 and CCG-203971 do not cause any detectable local toxicity or systemic side effects

**Figure 6:**
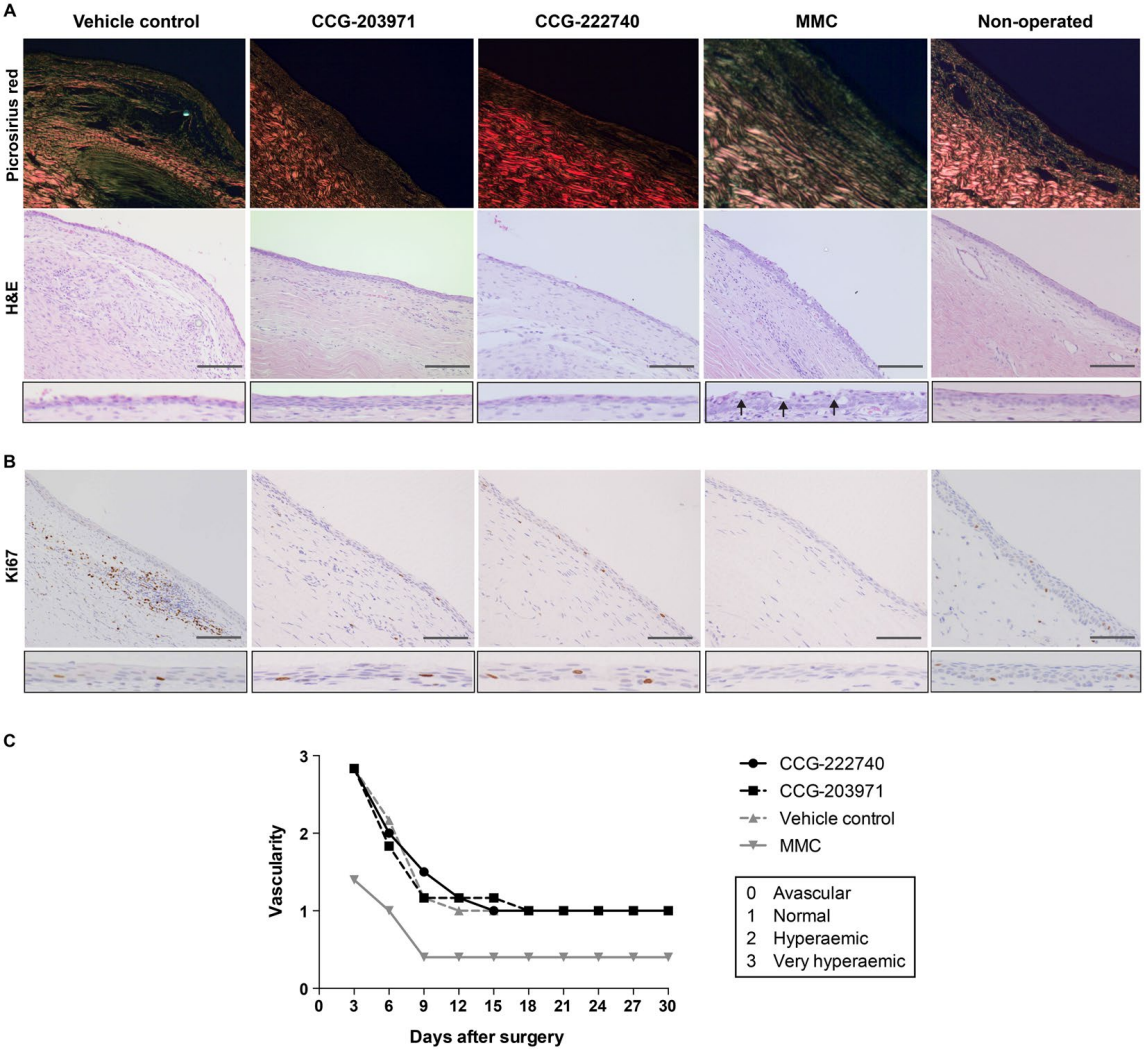
CCG-222740 and CCG-203971 preserve the conjunctival epithelium and do not cause any local toxicity. Sections were cut and stained as in Fig. 5. (**A**) H&E and Picrosirius red staining. Arrows indicate disruption of epithelium with MMC treatment. Scale bar = 100 μm. (**B**) Ki67 staining for proliferation. (**C**) Conjunctival vascularity as an indicator of local toxicity.

We also assessed the local toxicity and pharmacokinetics of CCG-222740 and CCG-203971. MMC exerts its anti-scarring effects by arresting the cell cycle and causing widespread apoptosis of fibroblasts in the conjunctiva^3^. However, the local toxicity of MMC is caused by the concurrent damage to the conjunctival epithelial layer leading to tissue breakdown and severe infection. MMC treatment led to characteristic thin and avascular blebs and caused disruption of the epithelial cell layer (Fig. 6A). We further stained the tissue sections for the Ki67 protein, which is a cellular marker of proliferation, and found that MMC treatment markedly reduced the number of Ki67 positive cells in the conjunctival epithelium (Fig. 6B). In contrast, CCG-222740 and CCG-203971 treatments did not cause any detectable local toxicity with an intact and healthy-looking conjunctival epithelium that was similar to the non-operated eyes, and with normal Ki67 positive cells in the conjunctival epithelium (Fig. 6A and B). There were also no signs of corneal or conjunctival toxicities or anterior chamber inflammation noted after local delivery of CCG-222740 or CCG-203971. Local tissue reaction to treatment was further assessed by conjunctival vascularity. MMC treatment led to thin avascular blebs but there were no abnormalities in conjunctival vascularity noted in the CCG-222740 and CCG-203971-treated groups (Fig. 6C).

In addition, the rabbits were carefully monitored throughout the study and none of them showed any signs of systemic toxicity after local delivery of CCG-222740 or CCG-203971 to the conjunctiva. We also tested the concentrations of CCG-222740 and CCG-203971 at day 30 in the aqueous, vitreous, and serum of the rabbits. Levels of CCG-222740 and CCG-203971 were very low and below 20 nM in all serum, aqueous, and vitreous samples collected 48 hours after the last dose was administered by local subconjunctival delivery.

## Discussion

There is mounting evidence that the MRTF/SRF pathway plays an important role in fibrosis as many key genes involved in fibrosis are also SRF target genes, including *ACTA2* and *CTGF* ^7, 35, 36^, and MRTF knockout models have shown reduced fibrosis in the heart^36^, lungs^37^, kidney^38^, and blood vessels^9^. Earlier MRTF-A inhibitors have proved efficient in targeting Rho-mediated MRTF/SRF signalling^14^, with a second generation inhibitor, CCG-203971, showing lower toxicity and increased efficiency^16, 17^. We show here that the novel MRTF/SRF pathway inhibitor CCG-222740 was more potent than the previously described CCG-203971 at decreasing MRTF/SRF target gene expression in human conjunctival fibroblasts, specifically expression of αSMA, a classical hallmark of fibrosis^39–41^, both *in vitro* and *in vivo*. Inhibition of MRTF/SRF signalling also prevented scar tissue formation in target tissues *in vivo*. MRTF/SRF pathway inhibition was not appreciably cytotoxic, but was as effective as traditional agents such as MMC at preventing scar tissue formation in a preclinical rabbit model of fibrosis.

Previous *in vivo* studies using the MRTF-A/SRF inhibitors CCG-1423^15^ and CCG-203971^16, 17^, established their anti-fibrotic potential in mouse models of vascular^9^, skin^10^, and lung fibrosis^11^. In all three studies, the inhibitors were injected intraperitoneally in mice, and were found to decrease fibrosis in blood vessels, skin, or lungs. The peak concentrations of inhibitor resulting from systemic injections in these studies were in a similar range to what we injected locally to the conjunctiva. Systemic delivery of compounds inhibiting the MRTF/SRF pathway nevertheless has potentially deleterious side effects in the body. MRTF plays a key role in vessel growth and maturation^42^, and endothelial depletion of murine MRTF/SRF can cause intracerebral haemorrhagic stroke^43^. Endothelial MRTF/SRF ablation can also lead to incomplete formation of the retinal vasculature in the eye^44^, and both SRF deletion or treatment with CCG-1423 significantly worsened denervation-induced muscle atrophy in mice^45^.

Given these concerns, we note that the eye has the advantage of being a closed compartment where small-molecule agents can be injected close to the target tissues, with minimal risks of systemic side effects^46, 47^. Local delivery of CCG-222740 and CCG-203971 did not cause any local or systemic adverse side effects in our study with very low drug levels measured in the aqueous, vitreous, and serum. This may reflect a rapid drug clearance, despite the blood-ocular barrier^48^, facilitated by the small drug quantities used as part of the targeted delivery. The blood-aqueous barrier and the blood-retina barrier both consist of cells joined together by tight junctions and maintain the eye as a privileged site for normal visual function^48^.

Mitomycin-C (MMC) is used to modulate wound healing in glaucoma surgery, to prevent recurrence in pterygium surgery, to prevent corneal haze in refractive surgery, and to treat conjunctival neoplasia^49^. However, MMC acts by inhibiting DNA synthesis and causes widespread apoptosis^3^, and serious vision-threatening side effects have been associated with its use, including hypotonous maculopathy^5^, severe infection^6^, corneal melting and perforation^50^, and scleral calcification^51^. In contrast, repeated administration of CCG-222740 or CCG-203971, which are relatively non-toxic^10^, was effective at reducing fibrosis and unlike MMC did not affect cell proliferation or induce apoptosis in the conjunctival epithelium. However, these compounds require a repeated dosing regimen to be effective, precluding their use in a clinical setting. Development of sustained-release formulations, or compounds with better pharmacokinetics, will thus be needed to take advantage of local drug delivery for successful translation to benefit patient care. In the absence of inhibitors with more favourable pharmacokinetics, future work will aim to successfully incorporate the current inhibitors into potential slow-release formulations, such as ocular tablet^32, 52^, microspheres^53^, microfilms^54^, or ocular implants^55^, for sustained drug delivery to the eye and other tissues.

While the effects of CCG-222740 and CCG-203971 are readily understood by their effect on MRTF/SRF signalling, the molecular target for these remains to be definitively established. There are currently two proposed mechanisms of action for the parent MRTF/SRF pathway inhibitor CCG-1423. Lundquist *et al*. have reported that CCG-1423 increases nuclear G-actin and decreases MRTF-A in the nucleus through inhibition of MICAL-2, a member of the MICAL family of actin-binding mono-oxygenases^18^. On the other hand, Hayashi *et al*. have reported that CCG-1423 acts directly on MRTF and other RPEL-family of proteins to prevent the nuclear import of MRTF-A^19^. In this regard, identifying the molecular targets of the new CCG-222740 described here could provide significant insight into the design of future therapeutic strategies. While CCG-222740 was clearly more potent than CCG-203971 in the contractility and gene transcription assays, the two compounds were equally efficacious in SRF reporter assays in transfected cells. The reasons for this remain unclear: it may reflect differential effects of the transfection protocol on subsequent cellular uptake of the two compounds, different sensitivities of authentic SRF genes to variations in inhibitor efficacy, or differential effects of the inhibitors on non-SRF targets.

This study provides proof-of-concept that local delivery of MRTF/SRF pathway inhibitors decreases scar tissue formation in a preclinical model of fibrosis. In particular, a novel MRTF/SRF pathway inhibitor, CCG-222740, is as effective as MMC in prolonging the long-term success of surgery but without detectable local or systemic adverse side effects. Although the optimum dose and drug delivery method of the inhibitor have yet to be determined, our results show that CCG-222740 could be delivered locally to target tissues to decrease scarring and fibrosis. Modulating the MRTF/SRF pathway using CCG-222740 thus represents a new avenue for regulating wound healing and for preventing post-surgical scarring in the eye and other tissues, with potential as a new and safe anti-fibrotic treatment in the future.

## Methods

### Patient sample and Animal use

Human conjunctival fibroblasts were isolated from donor eyes in the eye bank and informed consent was obtained from all subjects. All experimental protocols were approved by the institutional approval committee at the University College London Institute of Ophthalmology, and all the methods were carried out in accordance with the approved guidelines^56^. All animal procedures were also performed in accordance with the ARVO Statement for the Use of Animals in Ophthalmic and Vision Research, and all animal experimental protocols were approved by the Home Office UK (Project licence PPL 70/8074: maximising the success and effects of glaucoma surgery).

### Reagents and compounds

CCG-222740 and CCG-203971 were synthesised and characterised in the Vahlteich Medicinal Chemistry Core at the University of Michigan. Samples were >95% pure by high performance liquid chromatography (HPLC).

### Primary cell culture

We isolated primary cultures of human conjunctival fibroblasts from human conjunctival tissues. We also cultured rabbit conjunctival fibroblasts from New Zealand white rabbit conjunctival tissues. The tissues were mechanically dispersed and the tissue fragments were placed in tissue culture dishes in Dulbecco’s modified Eagle’s medium (DMEM, Invitrogen) with 10% fetal calf bovine serum (FBS), 100 U/ml penicillin, 100 mg/ml streptomycin, and 2 nM L-glutamine, in tissue culture incubators with 5% CO_2_ and 95% humidity. Fibroblasts between passages 2–8 were used in the experiments.

### Collagen contraction assay

We screened two candidate MRTF/SRF pathway inhibitors, CCG-222740 and CCG-203971, using detached three-dimensional fibroblast-populated collagen gels^56, 57^. Human or rabbit conjunctival fibroblasts were seeded in a collagen Type I matrix (First Link UK Ltd, Birmingham, UK) at a concentration of 0.7 × 10^4^ cells/ml. The fibroblast-populated collagen gel mixture was cast into the wells of MatTek dishes (MatTek Corp, MI, USA). The gels were detached from the edges of the well and 2 ml of growth medium were added to each well with 5, 25, 50, 75, or 100 μM of CCG-222740 or CCG-203971 or 0.1% DMSO control. Digital photographs were obtained daily and lattice areas were measured using Image J software (http://rsb.info.nih.gov/ij/). The gel size at day 7 was assessed as a percentage of lattice area on day 0. Each experiment was performed as triplicate gels per condition and the experiment was repeated on three separate days.

### SRF reporter gene assay

Human or rabbit conjunctival fibroblasts were plated at 2 × 10^4^ cells/well in 24-well plates (Falcon, Fisher Scientific) for transient transfection. Conjunctival fibroblasts in each well were co-transfected using 2.5 μl Lipofectamine LTX and 0.5 μl Plus reagent (Invitrogen) with 8 ng of SRF reporter plasmid 3DA.Luc, 20 ng of pTK-Renilla-Luc and 172 ng of pEF in OptiMEM (Gibco)^58^. After 2 hours of transfection, fibroblasts were allowed to recover in DMEM +10% FCS overnight.

HEK293T cells were plated at 2 × 10^4^ cells/well in 96-well plates (Falcon, Fisher Scientific) and co-transfected using Lipofectamine 2000 (Invitrogen) with 4 ng of SRF reporter plasmid SRE.L. luciferase^15^, and 2 ng of either G_α12_Q231L or pcDNA3.1/Zeo(−) diluted in Opti-MEM (Gibco). After 5 hours of transfection, cells were allowed to recover in DMEM +10% FCS overnight.

The next day, the cells were serum starved in DMEM +0.3% FCS and 5, 25, 50, or 100 μM of CCG-222740 or CCG-203971 or 0.1% DMSO control for 24 hours. The cells were then lysed and luciferase levels were measured using a Luciferase Assay System (Promega), according to the manufacturer’s instructions. Each experiment was carried out in triplicates for each condition and the experiment was repeated three times.

### Cell viability assay

Human conjunctival fibroblasts were plated at 6.25 × 10^3^ cells/well in 96-well plates (Falcon, Fisher Scientific), and treated with 1, 3, 10, 30 or 100 μM of CCG-222740 or CCG-203971 or 0.1% DMSO control for 24 hours. 20 μl of CellTiter 96 Aqueous one solution (Promega) were then added to each well containing the samples in 100 μl of culture medium. The plate was incubated for 1 hour at 37 °C and the absorbance was measured at 490 nm on a SpectraMax Plus 384 spectrophotometer (Molecular Devices, California, USA). Each experiment was carried out in triplicates for each condition and the experiment was repeated three times.

HEK293T cells were plated at 2 × 10^4^ cells/well in 96-well plates (Falcon, Fisher Scientific), and treated with 0.1, 0.3, 1, 3, 10, 30 or 100 μM of CCG-222740 or CCG-203971 or 0.1% DMSO control for 24 hours. Cell viability was measured using WST-1 reagent (10 μl/well, Roche) and read at 450 nm on a SpectraMax Plus 384 spectrophotometer (Molecular Devices, California, USA). The experiment was repeated three times.

### Real-Time quantitative PCR

Human or rabbit conjunctival fibroblasts were plated at 1 × 10^5^ cells/well in 6-well plates (Falcon, Fisher Scientific). The next day, the fibroblasts were treated with 5, 10, 25, or 50 μM of CCG-222740 or CCG-203971 or 0.1% DMSO control in DMEM +10% FCS for 24 hours. The fibroblasts were then lysed for RNA extraction using the Sigma RNA isolation kit (Sigma-Aldrich), according to the manufacturer’s instructions. Reverse transcription was carried out using the Transcriptor First Strand cDNA Synthesis Kit (Roche), according to the manufacturer’s instructions. qRT-PCR reactions were performed using SYBR green reagents (Life Technologies) on an HT7900 Fast Real-Time PCR system (Applied Biosystems). The human exonic primer sequences were: ACTA2, 5′-AATGCAGAAGGAGATCACGC-3′, 3′-TCCTGTTTGCTGATCCACATC-5′; CTGF, 5′-CAGAGTGGAGCGCCTGTT-3′, 3′-CTGCAGGAGGCGTTGTCA-5′; GAPDH, 5′-ACGGATTTGGTCGTATTGGGC-3′, 3′-TTGACGGTGCCATGGAATTTG-5′. The rabbit exonic primer sequences were ACTA2, 5′- TCCACCGCAAATGCTTCTAAGT-3′, 3′- ATGAGTCAGAGCTTTGGATAGGC-5′; GAPDH, 5′-CGAGACACGATGGTGAAGG-3′, 3′-CCAGCATCACCCCACTTGAT-5′. All mRNA values were normalised relative to that of GAPDH. Each experiment was carried out in triplicates for each treatment group and the experiment was repeated three times.

### Alpha smooth muscle actin staining

Human conjunctival fibroblasts were plated on 20 mm glass coverslips at 1 × 10^5^ cells/well in 6-well plates (Falcon, Fisher Scientific). The next day, the fibroblasts were treated for 48 hours with 10 or 25 μM of CCG-222740 or CCG-203971 or 0.1% DMSO control in DMEM +10% FCS. The samples were fixed in 4% paraformaldehyde for 10 minutes, washed three times in PBS, and permeabilised in PBS 0.2% TX-100 (Triton X-100, Sigma) for 20 minutes. The samples were blocked with 5% fetal calf serum/5% bovine serum albumin (Sigma) for 1 hour. The samples were incubated overnight with the Cy3-labelled alpha smooth muscle actin antibody (1:1000, Sigma) in a wet chamber at 4 °C in the dark. The next day, the samples were washed twice in PBS 0.1% TX-100 and incubated with phalloidin (1:200) and DAPI (1:2000) for 1 hour at room temperature. The samples were then mounted on slides and imaged using an Upright780 Zeiss LSM confocal microscope. The proportion of fibroblasts with prominent αSMA-positive stress fibres was quantified visually from three random fields of view per treatment group in 3 independent experiments.

### Western blotting

Human conjunctival fibroblasts were lysed using 2x SDS sample buffer (100 mM Tris HCL ph6.8, 4% SDS, 20% glycerol, 200 mM dithiothreitol, 0.2% bromophenol blue). Equal amounts of protein were loaded onto and run on 4–12% NuPAGE Bis-Tris protein gels (Novex, Life Technologies). The gels were transferred onto nitrocellulose blotting membranes (Amersham, Life Sciences), and blocked in 3% non-fat milk in PBST (PBS 0.1% Tween) for 1 hour. The membranes were then incubated overnight at 4 °C in αSMA primary antibody (1:200, polyclonal rabbit, ab5694, Abcam). The next day, the membranes were washed three times for 10 minutes in PBST, and incubated for 1 hour at room temperature in anti-rabbit HRP (1:2500, Dako). The membranes were then washed three times for 10 minutes in PBST, treated with ECL solution (Amersham, Life sciences) for 5 minutes, and scanned on an Odyssey IR Imager (LI-COR).

### Rabbit glaucoma filtration surgery model of scar tissue formation

24 female New Zealand white rabbits (2–2.4 kg, 12—14 weeks, Highgate, UK) underwent experimental glaucoma filtration surgery to the left eye under general anaesthesia^27, 28^. A superonasal fornix-based conjunctival flap was raised behind the limbus and a micro-vitreoretinal blade (20 gauge, 0.90 mm, Surgistar USA) was used to make a partial thickness scleral tunnel to the corneal stroma. A 22 gauge, 25 mm intravenous cannula was passed through the tunnel and the needle was removed once it was visible in the cornea. The cannula was advanced into the mid-pupillary area, trimmed at its scleral edge and fixed to the scleral surface using a 10–0 nylon suture (Alcon, USA). The conjunctival incision was closed using two interrupted 10–0 nylon sutures. In a randomised, prospective, single-masked observer study, the rabbits received either intraoperative 0.2 mg/ml mitomycin-C (MMC) [N = 6], or postoperative subconjunctival injections (100 μl) of 100 μM CCG-203971 [N = 6] or 100 μM CCG-222740 [N = 6] or vehicle control [N = 6] daily for the first nine days followed by twice weekly for three weeks^4, 33^. CCG-222740 and CCG-203971 were dissolved in 100% DMSO (Sigma, UK) at 10 mM and then diluted to 100 μM with sterile PBS. The same volume of DMSO was diluted with PBS for the vehicle control. For the MMC-treated group, a small corneal light shield sponge was soaked in 1 ml of 0.2 mg/ml MMC and applied over the conjunctiva for 3 minutes, and the area was then thoroughly washed with 50 ml of physiologic balanced salt solution (Alcon Laboratories, Inc., Fort Worth, TX). All the postoperative subconjunctival injections were given at the same site by a single-masked researcher.

### Post-operative clinical examination

The animals were evaluated twice weekly by a single-masked researcher. Bleb width and length were measured using calipers and intraocular pressures (IOP) were measured using a tonovet (Icare). The primary efficacy end-point was bleb survival as this is indicative of the long-term opening of the filtration pathway created during surgery. Bleb failure was defined as the appearance of a flat, scarred, and vascularised bleb associated with a deep anterior chamber. IOP survival, defined as when the intraocular pressure remains below the baseline IOP measurement, and bleb area were used as secondary end-points. Conjunctival vascularity was graded on a scale of 0 to 3 (0: avascular, 1: normal, 2: hyperaemic, 3: very hyperaemic).

### Histologic analysis

The animals were sacrificed on day 30 and both eyes were enucleated. The eyes were fixed in formalin, embedded in paraffin, and sequential 4 μm tissue sections were cut.

The sections were stained with hematoxylin and eosin (for cellularity and inflammatory cells), Gomori’s trichrome (for collagen), picrosirius red (for degree of fibrosis and collagen deposition), and alpha-smooth muscle actin (αSMA) using a primary monoclonal mouse anti-human αSMA antibody (Clone 1A4; Dako, High Wycombe, UK) and a biotinylated secondary antibody (rabbit anti-mouse; Dako). All the left operated eyes were compared to the right non-operated eyes that were used as controls for the appearance of normal conjunctival tissue. The sections were graded using a modified grading system originally described by Shah *et al*.^34^, with a scale of −4 to +4 according to the staining intensity. Grades were defined as 0 = same as control eye; 1 = 1–25% difference from control eye; 2 = 26–50% difference from control eye; 3 = 51–75% difference from control eye; 4 = >75% difference from control eye (prefix+, more than; prefix −, less than).

For the Ki67 staining, the sections were dewaxed, rehydrated, and microwaved for 15 minutes in sodium citrate buffer (pH 6) for antigen retrieval. After incubating in 1.6% hydrogen peroxide followed by 10% normal horse serum, the sections were incubated in mouse anti-Ki67 (M7248, Dako) diluted 1 in 25 in 1% BSA/PBS for 1 hour at room temperature. The sections were then incubated in biotinylated horse anti-mouse IgG (Vector) and Avidin-Biotin complex (Vector), developed in DAB (Vector), and counterstained in haematoxylin.

### Toxicity and Pharmacokinetics

All animals were carefully evaluated for any signs of ocular or systemic toxicity during the study. At day 30, 100 µl of aqueous, 1 ml of vitreous, and 1 ml of venous blood were sampled on the rabbit eyes receiving CCG-222740 or CCG-203971 or vehicle control. The venous blood samples were left to clot for 30 minutes at room temperature, spun at 4000 rpm for 15 minutes at 4 °C, and the serum was collected as the supernatant. Samples were extracted in acetonitrile and the levels of CCG-222740 and CCG-203971 were measured in the aqueous, vitreous, and serum using liquid chromatography/mass spectrometry (LC/MS) in the Metabolomics Core at Michigan State University (East Lansing, MI).

### Statistical analysis

Survival analyses for bleb and intraocular pressure were performed using the Kaplan-Meier log rank test. All graphs display mean and standard error of the mean (SEM). Statistical analysis was performed using the Student’s t-test to calculate statistically significant differences and individual *P* values. Statistically significant differences were expressed as *P < 0.05; **P < 0.01; ***P < 0.001.

## Electronic supplementary material

S1
