# Supplementary material for: Local delivery of novel MRTF/SRF inhibitors prevents scar tissue formation in a preclinical model of fibrosis
Source: Sci Rep. 2017 Mar 31;7:518. doi: 10.1038/s41598-017-00212-w (PMC5428058; doi:10.1038/s41598-017-00212-w)

**Local delivery of novel MRTF/SRF inhibitors prevents scar tissue formation in a preclinical model of fibrosis**

### Cynthia Yu-Wai-Man^1, 2, 3^ *, Bradley Spencer-Dene^4^, Richard M.H. Lee^2^, Kim Hutchings^5^, Erika M. Lisabeth^6^, Richard Treisman^3^, Maryse Bailly^1^, Scott D. Larsen^5^, Richard R. Neubig^6^, Peng T. Khaw^2^

1. Department of Cell Biology, UCL Institute of Ophthalmology, London, United Kingdom.
2. National Institute for Health Research (NIHR) Biomedical Research Centre at Moorfields Eye Hospital NHS Foundation Trust and UCL Institute of Ophthalmology, London, United Kingdom.
3. Signalling and Transcription Group, Francis Crick Institute, London, United Kingdom.
4. Experimental Histopathology STP, Francis Crick Institute, London, United Kingdom.
5. Vahlteich Medicinal Chemistry Core, College of Pharmacy, University of Michigan, Ann Arbor, MI, USA.
6. Department of Pharmacology and Toxicology, Michigan State University, East Lansing, MI, USA.

**Supplementary figure S1.** Blots from Fig. 3


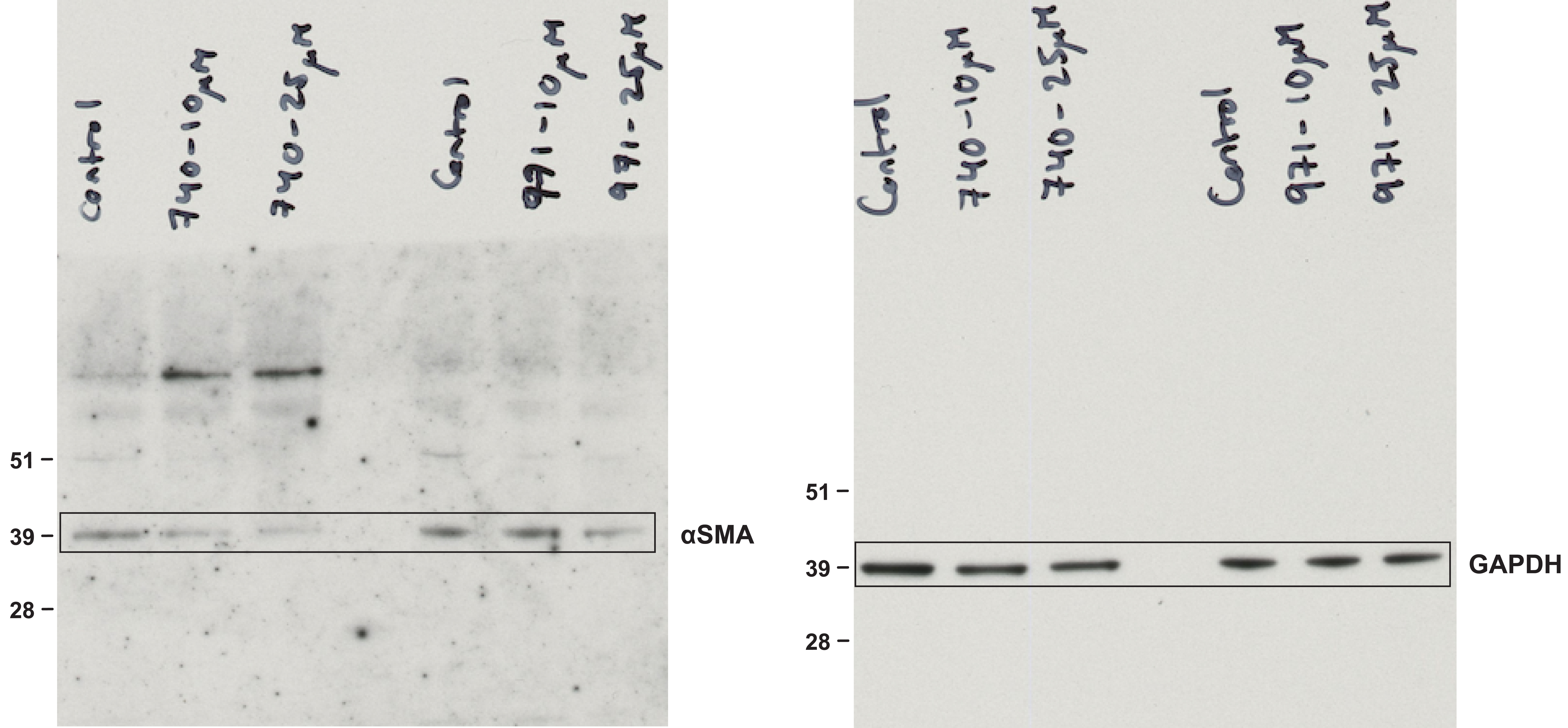

Supplement: Supplementary file 1 — S1 [file 41598_2017_212_MOESM1_ESM.doc]
